# Cardiovascular disease and depression: a bidirectional relationship and its clinical implications

**DOI:** 10.3389/fpsyt.2025.1675680

**Published:** 2026-01-12

**Authors:** Jianbo Wu, Zifan Tian, Zhimin Qi, Xiaoqun Liu, Peng Yu

**Affiliations:** 1 Department of Endocrinology and Metabolism, The Second Affiliated Hospital of Nanchang University, Nanchang, Jiangxi, China; 2 Huan Kui College of Nanchang University, Nanchang, China; 3 Department of Anesthesiology, School of Stomatology, Jiangxi Medical College, Nanchang University, Nanchang, China; 4 Department of Respiratory and Critical Care, School of Clinical Medicine, Jiujiang University, Jiujiang, Jiangxi, China

**Keywords:** CVD, depression, bidirectional relationship, treatment strategies, blood-brain barrier

## Abstract

There is strong evidence that depression is linked to greater rates of morbidity and death in people with cardiovascular disease (CVD), supporting its status as a key risk factor for the condition. Recent researches suggest a reciprocal relationship, with CVD potentially predisposing people to depressive disorders. Notably, depression has a high comorbidity rate with major cardiovascular disorders such as coronary artery disease and myocardial infarction, which may have a severe impact on patients’ long-term outcomes. Several pathways, including neuroendocrine dysregulation, activation of the inflammatory system, and behavioral alterations, appear to underlie the connection between depression and CVD. On the other hand, CVD may lead to the pathophysiology of depression by altering brain hemodynamics and causing blood–brain barrier (BBB) damage. Current therapeutic approaches stress a multimodal approach that combines drug interventions, cognitive behavioral therapy, and lifestyle changes to improve patient results. This review summarizes the latest research findings on the complex connection between depression and cardiovascular diseases, as well as contemporary treatment options and clinical consequences.

## 1 Introduction

Depression and Cardiovascular Disease (CVD) are serious global health burdens that affect the lives of millions of people. Depression, a common mental health disorder, affect the mental health and quality of life of over 350 million individuals worldwide. Due to its chronicity, high prevalence and recurrent episodes, depression is projected to be the leading cause of disability globally by 2030 (1, 2). CVD, on the other hand, are one of the main causes of reduced average life expectancy, with high morbidity (3). The social burden of CVD has been rising for decades and, worryingly, the age-standardized prevalence of CVD is increasing in some regions, whereas it was previously on the decline in high-income countries (4).

Increasing evidence highlights the bidirectional relationship between depression and CVD. Key healthcare and economic indicators related to both conditions, such as growing healthcare expenses, increased healthcare utilization, and productivity losses, underscore their substantial impact. Both depression and CVD can profoundly affect overall quality of life. Individuals with mood disorders, including bipolar disorder and major depressive disorder (MDD), are shown in meta-analyses to have an increased risk of coronary artery disease (CAD), cerebrovascular disease, congestive heart failure, and cardiovascular mortality, with an odds ratio as high as 2.1 (5). Moreover, MDD is particularly prevalent among individuals experiencing acute cardiac events, with up to 40% of patients meeting the diagnostic criteria for MDD (6). Among CAD patients, more than 14% are diagnosed with depression (7). Additionally, depression is known to increase the risk of cardiovascular death in both the general population and those who already have heart disease. According to a meta-analysis, depression raised the chance of dying from cardiovascular disease by 63% (8). Furthermore, depression is linked to a heightened risk and poorer prognosis for other cardiovascular conditions, including peripheral artery disease, stroke and heart failure (9, 10).

Therefore, a profound exploration of the relationship between the cardiovascular system and depression is essential for the prevention, diagnosis, and treatment of these two interrelated disorders.

## 2 Methods

This narrative review aimed to synthesize current evidence on the bidirectional relationship between depression and CVD, focusing on underlying mechanisms, clinical implications, and treatment strategies. To ensure a comprehensive and up-to-date overview, a systematic literature search was conducted.

### 2.1 Review design and search strategy

We performed literature searches in two major databases: PubMed and Google Scholar. The search was limited to articles published between 01-01–2005 and 06-30–2025 to capture the evolving understanding over the past two decades, including recent advances. The primary search terms included: “depression,” “major depressive disorder,” “cardiovascular disease,” “CVD,” “bidirectional relationship,” “mechanisms,” “HPA axis,” “inflammation,” “autonomic nervous system,” “blood-brain barrier,” “treatment,” “SSRIs,” “cognitive behavioral therapy,” and “vagus nerve stimulation.” These terms were used in various combinations to maximize retrieval of relevant studies.

### 2.2 Inclusion and exclusion criteria

Studies were included if they: 1) were original research articles (including randomized controlled trials, prospective and retrospective cohort studies, case-control studies, and meta-analyses) or authoritative reviews; 2) investigated the association between depression and CVD, explored potential pathophysiological mechanisms (e.g., neuroendocrine, inflammatory, autonomic), or evaluated treatment efficacy and safety in comorbid populations; 3) were published in English. Articles were excluded if they: 1) were editorials, letters, or case reports with limited generalizability; 2) did not have the full text accessible.

### 2.3 Study selection and data extraction

The literature search and selection process were conducted by the authors. Initially, titles and abstracts of retrieved articles were screened for relevance. Potentially eligible full-text articles were then assessed against the inclusion criteria. Key data extracted from the selected studies included: study design, sample characteristics, main findings related to depression-CVD interplay, mechanistic insights, and clinical outcomes. Given the narrative nature of this review, a formal quality assessment or risk-of-bias tool (such as the Cochrane RoB tool for RCTs or Newcastle-Ottawa Scale for observational studies) was not systematically applied to all included studies. However, particular attention was paid to the methodological rigor of the cited works, especially large-scale meta-analyses, prospective cohorts, and recent high-impact publications (2024-2025), to ensure the reliability of the conclusions drawn. The synthesis of evidence was thematic, organizing findings into logical sections covering the effects of depression on CVD and vice versa, culminating in clinical implications.

## 3 The effect of depression on cardiovascular system

### 3.1 Neuroendocrine system

#### 3.1.1 Physiological mechanisms and normal rhythms of cortisol secretion

Cortisol in the human body is the predominant glucocorticoid hormone, the production and secretion of which is regulated by the hypothalamic-pituitary-adrenal (HPA) axis (11). Under normal physiological conditions, cortisol levels are relatively low, exhibiting a circadian rhythm. In general, cortisol levels rise rapidly upon awakening, a rise known as the cortisol awakening response (CAR). This is followed by a gradual decrease throughout the day, usually to a minimum shortly after people go to sleep (12). During the night, cortisol concentrations are approximately half of those during the daytime, with normal values ranging from 110 to 390 nmol/L, with the lowest values occurring most often around 4:00 a.m (13, 14). In addition to the circadian rhythm, cognitive and neurogenic stress signals also influence cortisol secretion. In response to stress, the hypothalamus produces corticotropin-releasing hormone (CRH), which prompts the anterior pituitary gland to secrete adrenocorticotropic hormone. This sequence of events ultimately leads to the production and release of cortisol from the adrenal glands (13).

Mineralocorticoid receptors and glucocorticoid receptors are the two main receptors for glucocorticoid action on the HPA axis. Cortisol binds to these receptors to induce various critical functions in the body, including the regulation of immune responses and stress responses (15). As part of the body’s defense mechanism, cortisol attenuates inflammatory responses and stimulates gluconeogenesis to prevent excessive immune reactions. Under stress conditions, cortisol enhances energy metabolism by increasing gluconeogenesis and promoting insulin resistance, thereby facilitating a more efficient stress response (16). Additionally, cortisol regulates blood pressure and vascular tone, leading to vasoconstriction and increased cardiac output, which are essential for the acute stress response (17).

#### 3.1.2 The impact and mechanisms of depression on cortisol secretion

Compared to healthy individuals, depressed individuals exhibited higher mean daytime cortisol levels and were more pronounced on the CAR (18), indicating that depressed individuals secrete more cortisol during the day and in the morning peak periods. Moreover, individuals at risk for depression show notably higher morning cortisol levels compared to the healthy individuals (19). A Study also demonstrate a significant positive correlation between serum cortisol levels and Hamilton Depression Rating Scale scores, further supporting the notion of dysregulated cortisol secretion in depression (20). Research suggests that elevated morning cortisol levels precede the onset of MDD during adolescence, whether it is a first episode or a recurrence (19). This finding implies that increased cortisol levels may serve as a predictor of depression, rather than a pathological consequence. Longitudinal studies have shown that higher morning cortisol levels can predict depressive episodes, and prospective research by Adam et al. confirms that a more pronounced CAR significantly predicts the onset of depression (21). The initial elevation of cortisol levels, which most likely stems from the individual’s exposure to stressful situations, is a precursor to the subsequent development of MDD. As stress exposure accumulates, the cortisol response diminishes, and MDD develops as the individual reaches puberty (19, 22).

Unlike healthy individuals, patients with depression exhibit heightened sensitivity to daily stressors and greater emotional instability, which may cause fluctuations in cortisol levels (23). The response of depressed individuals to external stressors differs from that of healthy controls, leading to cortisol release. Acute stress causes a transient increase in cortisol levels, which does not have long-term effects on health (24). However, chronic stress disrupts the normal rhythmic secretion of cortisol. Chronic stress exposure disrupt CB1 receptor signaling in the amygdala, which ultimately activate the HPA axis through neurotransmitter release in the prefrontal cortex and hippocampus (25). It has also been shown that dysfunction of medial hypothalamic neurons, which are responsible for inhibiting the secretion of CRH and pressor hormones, may lead to excessive cortisol release (26, 27).

#### 3.1.3 Impact of abnormal cortisol secretion patterns on the cardiovascular system

Cortisol affects cardiovascular tissue through multiple mechanisms. It inhibits the secretion of PGI2 from endothelial cells by down-regulating the expression and activity of enzymes involved in the synthesis of PGI2, an important vasodilator and inhibitor of platelet aggregation. In turn, continuous infusion of PGI2 analogues induces hypopituitarism (28). This mechanism may underlie the ability of cortisol to increase blood pressure and enhance vascular reactivity. In addition, cortisol also stimulates platelet activation and aggregation, leading to surface exposure of CD62P and an increase in intracellular calcium. Cortisol enhances its aggregation effect and reduces the level of the potent anticoagulant NO. Through these mechanisms, cortisol enhances its ability to induce oxidative stress in human platelets (29).

**Figure 1:**
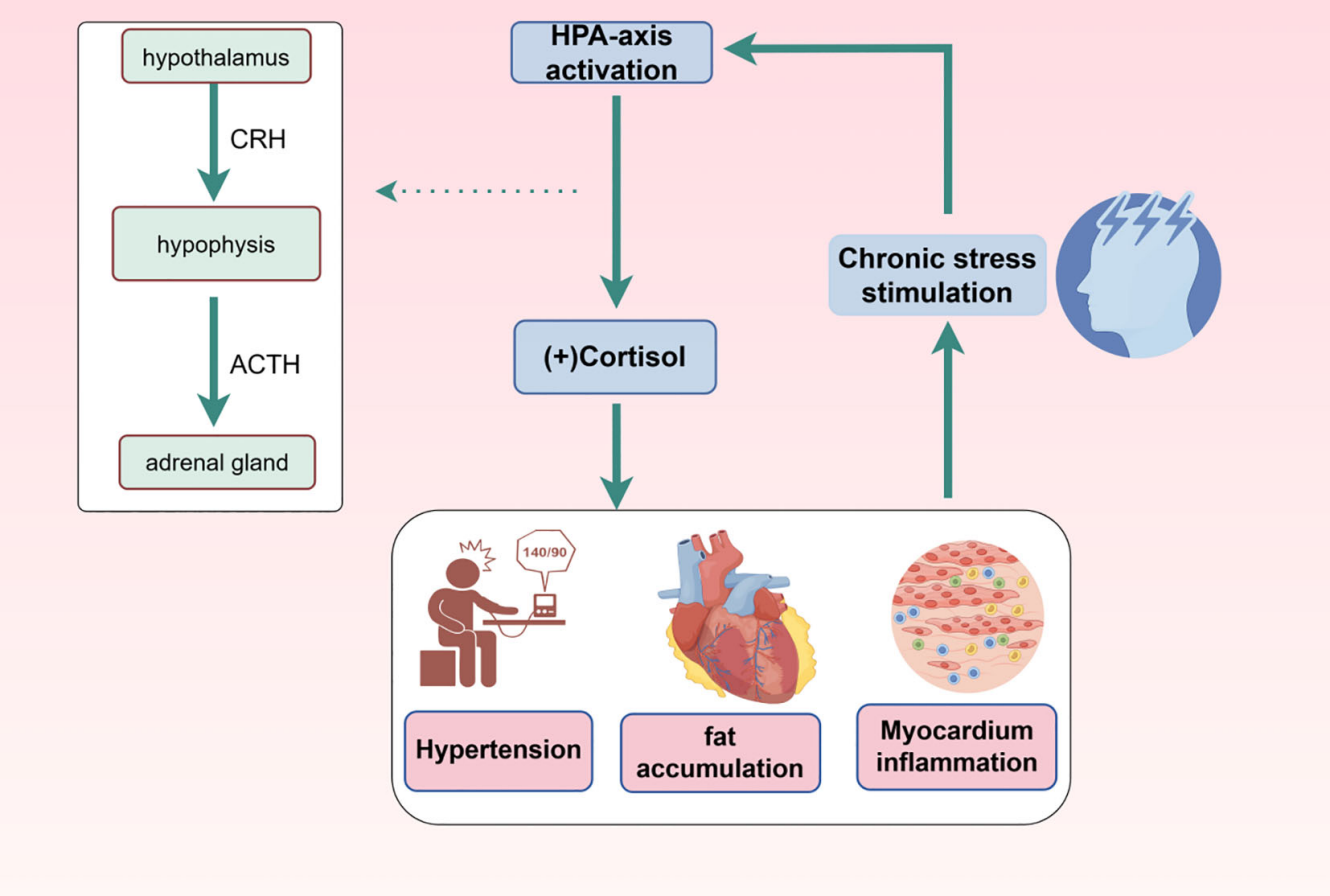
Effects of HPA axis activation on cardiovascular function and worsening of CVD by chronic stress. Chronic stress accumulation can cause activation of the HPA axis and increased cortisol secretion, which in turn can lead to consequences such as high blood pressure, cardiac fat accumulation, and myocardial inflammation. In turn, these consequences may cause chronic stress in the organism.

Excessive cortisol secretion is associated with several cardiovascular adverse events (**Figure 1**), including hypertension, central obesity, hyperinsulinemia, hyperglycemia, and dyslipidemia. Cortisol exacerbates visceral fat formation by inhibiting the growth hormone and gonadal axes, adding to dyslipidemia, and, combined with hypercortisolemia, promotes the development of insulin resistance and hyperinsulinemia (30, 31). In the context of CVD, elevated cortisol levels have been shown to increase the risk of complications and mortality in patients with acute myocardial infarction (32). High cortisol levels are also associated with larger myocardial infarction areas, ventricular remodeling after acute myocardial infarction, and higher mortality rates in chronic heart failure patients (33). Experimental evidence indicates that persistent elevated cortisol levels generate mild myocardial inflammation, defined by an increased number of neutrophils and lymphocytes, resulting in cardiac dysfunction and arrhythmias (34). Additionally, long-term cortisol dysregulation due to extreme emotional stress may trigger ventricular fibrillation and sudden cardiac death.

Most cases of sudden cardiac death are accompanied by ventricular fibrillation and underlying inflammation caused by high cortisol level (35), suggesting that high circulating cortisol concentrations may create focal or systemic inflammation, causing the onset of sudden cardiac death. Cortisol has also been recognized as an independent prognostic marker of function in daily living and mortality in patients with acute ischemic stroke (36). Hypercortisolism is associated with more severe neurological damage, larger ischemic lesions, and poorer outcomes in stroke patients. Furthermore, cortisol can modulate vascular reactivity. Higher cortisol levels increase arterial responsiveness to norepinephrine-induced contraction and inhibit platelet aggregation, increasing the risk of atherogenesis (37, 38).

#### 3.1.4 Autonomic dysregulation in depression

The autonomic nervous system (ANS) regulates heart rate through the intrinsic cardiac pacemaker, while heart rate variability (HRV) serves as an indicator of the equilibrium between the sympathetic and parasympathetic branches of this system (39). HRV is commonly used in clinical settings as a physiological marker of autonomic function, with reduced HRV indicating abnormal sympathetic activation (40). Chronic stress experiments have demonstrated that depression is associated with reduced HRV, increased resting heart rate, and heightened sympathetic nervous activity (41). Data from one study showed a significantly lower HRV index in depressed patients, indicating diminished cardiac vagal activity in depressed patients (42). An analysis of children and adolescents that included 31 articles found that depression severity was significantly and negatively correlated with high frequency (r = -0.10) and root mean of the square of the consecutive differences (r = -0.18) in HRV (43). Furthermore, levels of norepinephrine in the cerebrospinal fluid and plasma are significantly elevated in depressed patients (44). These findings suggest that autonomic dysregulation, characterized by increased sympathetic activity, persists during depression. SSRIs have been shown to reduce sympathetic nervous activity in both the body and heart, as evidenced by improvements in HRV and a decrease in the mean daily heart rate (45). This effect may be mediated through the alleviation of depressive symptoms, or alternatively, through SSRI inhibition of sympathetic signaling at the medullary level. Additionally, non-anticholinergic antidepressants and cognitive therapy can reverse the increase in sympathetic activity associated with depression (45), further suggesting that enhanced sympathetic activity is a consequence of depression, rather than its cause. In addition to the overactivation of the sympathetic nervous system, vagal (parasympathetic) activity, which counteracts sympathetic effects on the heart, is also suppressed. Research indicates a notable inverse relationship between psychosocial functions and vagal nerve activity (46). Research findings suggest that patients with MDD exhibit notably reduced levels of parasympathetic markers, including the Valsalva ratio, the standing-to-supine ratio, and results from deep breathing assessments, when compared to healthy individuals (47), indicating reduced parasympathetic responsiveness in depression. In conclusion, the dysregulation of the balance between sympathetic and parasympathetic nervous system activity is closely associated with depression. In clinical practice, vagus nerve stimulation (VNS) may be used as a treatment for depression. Mechanistically, VNS may exert antidepressant effects by affecting neurotransmitters. Studies have shown that VNS modulates the release of monoamine neurotransmitters such as norepinephrine and 5-hydroxytryptamine, which are closely related to the development of depression (48). VNS is able to affect neuroplasticity in the brain, especially in the hippocampal region. The hippocampus is closely related to emotion regulation, and VNS may ameliorate depressive symptoms by increasing hippocampal gray matter volume, which also suggests that hippocampal remodeling may be a marker of VNS response (49). In addition, VNS may activate brain regions associated with emotion, such as insular cortex, and amygdala (50), the activation of these regions may be related to the antidepressant effects of VNS.

#### 3.1.5 Impact of autonomic nervous dysfunction on cardiovascular health

The entanglement between depression and clinical cardiovascular events may be caused by autonomic nervous system (ANS) dysfunction. Abnormalities in sympathetic nerve activity have been suggested as one possible mechanism for this association (51). Sympathetic overactivation is an essential cause of cardiovascular dysfunction and endothelial injury. Norepinephrine dilates coronary arteries (prostaglandin-mediated dependence) while constricting femoral arteries. However, during atherosclerosis, coronary prostaglandin biosynthesis is inhibited, which may impair its protective diastolic function (52). Experimental evidence has demonstrated that the increased vascular tone observed in the morning is attributed to enhanced α-adrenergic vasoconstriction, which is a risk factor for various adverse cardiovascular events, such as hypertension. Additionally, studies in high-altitude populations have shown that chronic hypoxia-induced elevated α-adrenergic activity, which leads to reduced endothelium-dependent vasodilation, further impairing endothelial function (53). Further studies have shown that depression accompanied by sympathetic activation promotes vascular inflammation and atherosclerosis through mechanisms such as reduced endothelial cell production and brachial artery blood flow and restricted vasodilation (54). β-adrenergic blockers inhibit sympathetic nerve activity and have been shown to reduce myocardial infarction events during the morning peak period (55, 56), indirectly supporting this hypothesis. Reduced HRV, another marker of sympathetic activity, has been shown to predict the onset of sudden cardiac death, as low HRV is observed prior to the onset of sudden death. Moreover, depressed individuals with low HRV also exhibit impaired cardiopulmonary vascular reactivity (57). Taken together, ANS dysfunction may play a crucial role in the adverse cardiovascular effects of depression.

Sympathetic nervous activity is also associated with sustained increases in blood pressure, through mechanisms including increased peripheral vascular resistance and cardiac contractility, reduced venous capacitance, and promotion of renal sodium and water retention (58). Study has shown that patients with diabetes exhibit abnormalities in their blood pressure circadian rhythm, characterized by reduced nighttime blood pressure dipping, with the degree of circadian disruption positively correlated with the frequency of nighttime sympathetic activity (59). Furthermore, high sympathetic outflow is associated with increased daytime blood pressure variability and significant nighttime blood pressure reduction in healthy individuals (60, 61). These findings suggest that high sympathetic activity leads to blood pressure dysregulation, increasing the risk of hypertension-related conditions such as stroke.

Compared to healthy controls, individuals with depression are more prone to arrhythmias, including atrial fibrillation, bradycardia, supraventricular arrhythmias, and ventricular arrhythmias (62). Disruption of the balance between sympathetic and parasympathetic nerves as a possible pathway for arrhythmia caused by depression (51, 63). Sympathetically driven arrhythmias are diverse, ranging from severe ventricular tachycardia in pathological conditions to occasional supraventricular and ventricular ectopic beats in healthy hearts. Mechanistically, excessive activation of the sympathetic nervous system leads to abnormal adrenergic activation, increasing rhythmic activity in ventricular Purkinje fibers and shortening the diastolic phase, thereby predisposing the heart to ventricular arrhythmias (64). Additionally, increased nocturnal sympathetic activity has been shown to promote arrhythmogenesis through elevated blood pressure and vasoconstriction (65).

### 3.2 Inflammation

#### 3.2.1 Sources and effects of cytokines

Cytokines are mostly produced by immune cells and play a role in the promotion or suppression of inflammation. Cytokines such as IL-1β, IL-6, IL-12, IL-17, TNF-α, TNF-β, and IFN-γ exhibit pro-inflammatory effects, while IL-10 and IL-4 exert anti-inflammatory effects (66). IL-6, secreted by Th cells, exerts an effects on B cell proliferation and differentiation through IL-6 receptors or gp130 signaling (67). TNF-α is produced by macrophages, mast cells, and natural killer cells, stimulating macrophages to release pro-inflammatory cytokines and inflammatory mediators (68). ELISA is widely used for the detection of various biomarkers, including levels of inflammatory factors, because of its ease of use, sensitivity, and specificity. In studies, ELISA was used to analyze cytokines, such as IFN-γ, in order to investigate their correlation with changes in depressive symptoms (69). ELISA was also used to study the production of TNF-α triggered on monocytes by ApoB binding to ENO1 (70). PCR technology has also been used to evaluate inflammatory markers such as hs-CRP, IL-6 with good specificity and reproducibility (71).

#### 3.2.2 Cytokine changes induced by depression

Patients with depression exhibit significant inflammatory characteristics, particularly in peripheral blood, where levels of inflammation-related factors and their receptors, such as IL-1β, IFN-γ, chemokines (e.g., CCL2, CXCL4, CXCL7), and other inflammatory mediators, are markedly elevated (72). In addition, acute phase proteins including TNF-α, CRP and IL-6 are also increased (73–75). In the brains of patients with depression, elevated levels of pro-inflammatory factors and the activation of their signaling pathways have also been observed (76). Typically, these increases in inflammatory factors should not occur in the absence of infection or injury, indirectly suggesting a potential link between depression and inflammation. Depression is often associated with dysfunction of both innate and adaptive immune systems, which not only affects immune responses but may also hinder favorable prognosis (66). Furthermore, the expression of inflammation-related genes in peripheral blood mononuclear cells (PBMCs) from patients with depression has been noted to be elevated (77).

A large-scale meta-analysis indicated that levels of TNF-α, IL-6, IL-1RA are elevated in patients with depression, while IFN-γ levels tend to decrease (72). Several studies have shown that multiple inflammatory factors are abnormally elevated in depression, which may reflect an immunological manifestation of the disorder. Supporting evidence includes a longitudinal study that found no direct association between CRP levels and subsequent depressive states (78); however, cumulative depressive episodes independently predicted later CRP levels. Moreover, high levels of IL-6 were associated with a rapid progression of depressive symptoms in patients, and TNF-α was also linked to worsening depression (79).

In rodent stress models, Infiltration of peripheral monocytes into the brain is accompanied by an increase in inflammatory factors in the brain and depressive-like behavior (80), suggesting that monocytes may be a major source of inflammatory factors in depression. Other studies have indicated that perfusion defects associated with depression may activate microglia, thereby triggering neuroinflammation in older populations (81). On the other hand, inflammatory factors may further exacerbate the development of depression. Hippocampal neurogenesis has a place in the pathophysiology of depression, and one of the effects of SSRIs is to alleviate symptoms by promoting the proliferation of hippocampal neural progenitor cells (82, 83). A study has found that IL-6 acts as an anti-neurogenic signal in microglia, inhibiting the activity of neural progenitor cells, while TNF-α also has a significant anti-proliferative effect on these cells (84). In addition, Inflammatory factors also decrease the utilization of serotonin and melatonin precursors and activate the kynurenine pathway of the neurotoxic metabolite quinolinic acid, which in turn causes depression (85, 86).

#### 3.2.3 Cardiovascular consequences of cytokine dysregulation

Inflammatory mediators such as IL-6, TNF-α, and IL-1β play a crucial role in cardiovascular disease, particularly in the pathogenesis of atherosclerosis, endothelial dysfunction, and CVD. These pro-inflammatory cytokines affect cardiovascular health through various mechanisms. For instance, IL-6 regulates endothelium-dependent vasodilation, monocyte differentiation, platelet function, and endothelial prothrombotic states (87). IL-6 is produced by a range of cells, including macrophages and endothelial cells, and its expression is significantly upregulated under conditions such as inflammation, angiotensin II (Ang II) stimulation, oxidative stress, and vascular injury (88, 89). Studies have shown that IL-6 directly impacts endothelial cells, inducing the production of various inflammatory cytokines and chemokines, thereby activating the coagulation cascade, leading to coagulopathy and vascular leakage (90).What’s more, IL-6 boosts the activity of the AT1R gene in endothelial cells, which could drive Ang II-induced vasoconstriction and the production of ROS, a major contributor to endothelial dysfunction (91). Studies show that IL-6 exposure heightens platelet sensitivity to thrombin and ramps up the expression of activation markers like P-selectin (92). In COVID-19 patients, elevated levels of IL-6 were associated with significant upregulation of P-selectin and PSGL-1, suggesting that IL-6 may exacerbate immune thrombosis by promoting platelet-leukocyte aggregation and endothelial activation (93).

**Figure 2:**
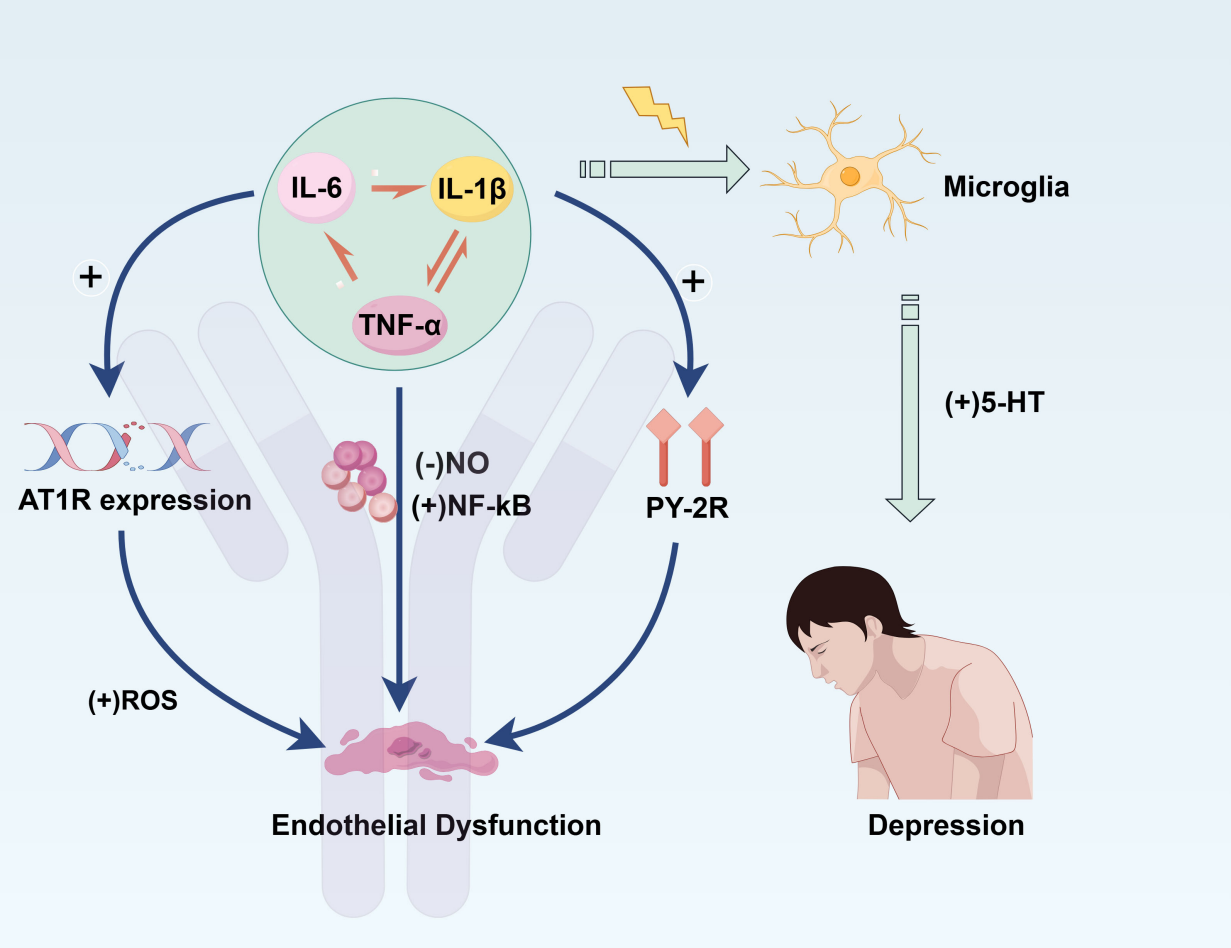
The pro-inflammatory factors IL-6, IL-1β and TNF-α cause endothelial cell disorders through different pathways, respectively. These pro-inflammatory factors activate microglia and promote 5-HT secretion to influence depression.

In addition to IL-6, other proinflammatory mediators such as IL-1β and TNF-α also participate in vascular pathology (**Figure 2**). Studies have shown that IL-1β can accelerate the progression of atherosclerosis by inducing lesions in vascular smooth muscle cells through upregulation of P2Y receptors (94). As another potent inflammatory cytokine, TNF-α promotes the expression of inflammatory mediators and regulates endothelial-dependent vasodilation. Research indicates that TNF-α destabilizes eNOS mRNA, hindering NO synthesis, while enhancing the levels of various inflammatory cytokines and chemokines, such as IL-6, NF-κB, and AP-1 (95). This TNF-α-mediated signaling pathway accelerates thrombosis, vascular remodeling, endothelial cell apoptosis, and vascular oxidative stress, all of which are closely associated with endothelial dysfunction (96). There are interactions between inflammatory factors. In certain inflammatory states, elevated IL-6 levels can promote TNF-α production (97). In addition, TNF-α is also capable of stimulating IL-6 expression, creating a positive feedback mechanism that leads to further exacerbation of the inflammatory response (98). Research indicates that IL-6 and TNF-α work together to inflict damage on endothelial cells, which can result in vasodilatory dysfunction and destabilization of atherosclerotic plaques (99). IL-6 secretion responds to the combined processing of TGF-β and IL-1β, suggesting that IL-1β may interact with IL-6 and participate in the inflammatory process (100). Additionally, targeted interventions aimed at the inflammasome-IL-1β-IL-6 pathway have demonstrated promising results in lowering cardiovascular incidents, underscoring the significance of the interplay between IL-1β and IL-6 in inflammation (101). There is also evidence suggesting synergistic effects between IL-1β and IL-6. Diabetic mouse models show that upregulation of the ERK/phosphorylated p65/IL-1β/IL-6/TNF-α pathway impairs vascular endothelial cell tube formation and migration capacity (102). Furthermore, TNF-α promotes ROS production by activating NADPH oxidase, thereby disrupting the dynamic balance of vasodilation and vasoconstriction (103).

### 3.3 Bad lifestyle

People with depression often exhibit unhealthy habits, including poor eating habits, sleep disorders and reduced exercise.

Research has shown that dietary patterns in individuals with depression tend to change. In patients with depression, the intake of fish, vegetables, nuts, and whole grains is lower than that of healthy individuals (104). Regular consumption of these foods is often linked to a reduced risk of CVD and associated risk factors (105). Thus, a healthy diet reduces the risk of cardiovascular disease. Regarding appetite, there is marked heterogeneity among depressed patients, with some experiencing reduced appetite and others increased appetite. In melancholic depression, significant anorexia and/or weight loss are typically observed, whereas individuals with atypical depression often experience increased appetite and/or weight gain (106). Appetite disturbances in depressive patients may be related to cytokine-induced anorexigenic leptin release (107). In addition, the depressed group with increased appetite had increased blood oxygen level-dependent (BOLD) responses in brain associated with the reward system. In contrast, those with decreased appetite often exhibit reduced BOLD activity in the posterior insula, which may explain different patterns of appetite dysregulation in depression from a central nervous system perspective (108). Dietary interventions for people with depression include reducing the intake of foods high in sugar and fat and increasing the intake of foods rich in omega-3 fatty acids and multivitamins, which may improve symptoms of depression (109, 110).

Sleep disturbances are also common symptoms of depression. Ninety percent of patients with major depression reported sleep disturbances compared to the healthy group (111). Irregular sleep patterns lead to disrupted circadian rhythms, increasing the incidence of major cardiovascular events (112). Studies have shown that shift workers, with disrupted circadian rhythms, face a significantly higher cardiovascular risk (113). Additionally, circadian misalignment can directly cause dyslipidemia and vascular inflammation (114), which may exacerbate or trigger CVD. In a prospective analysis, greater variability in sleep duration was linked to an increased prevalence of CVD and metabolic disorders such as diabetes. Moreover, short-term circadian disruption increases 24-hour blood pressure levels by raising glucose, cholesterol, and triglyceride levels, while promoting the release of systemic inflammatory markers such as IL-6, CRP, and TNF-α, potentially further increasing cardiovascular risk (115–117). Cognitive Behavioral Therapy (CBT) is considered an effective treatment for insomnia, especially for patients with CVD. CBT improves the quality of sleep by altering patterns of thought and behavior that affect sleep. In patients with CVD, CBT-I (Cognitive Behavioral Therapy for Insomnia) improves symptoms of insomnia and fatigue and may have an effect on sleep-related cognitions, and these cognitive changes are associated with improvements in sleep and symptoms (118). Not only does CBT improve insomnia symptoms, it may also positively affect CVD risk factors such as anxiety and depression (119), and improvements in these risk factors may lead to a lower incidence of CVD. In addition, therapies such as physical activity and continuous positive airway pressure ventilation are also beneficial to the prognosis of patients with CVD (120).

**Figure 3:**
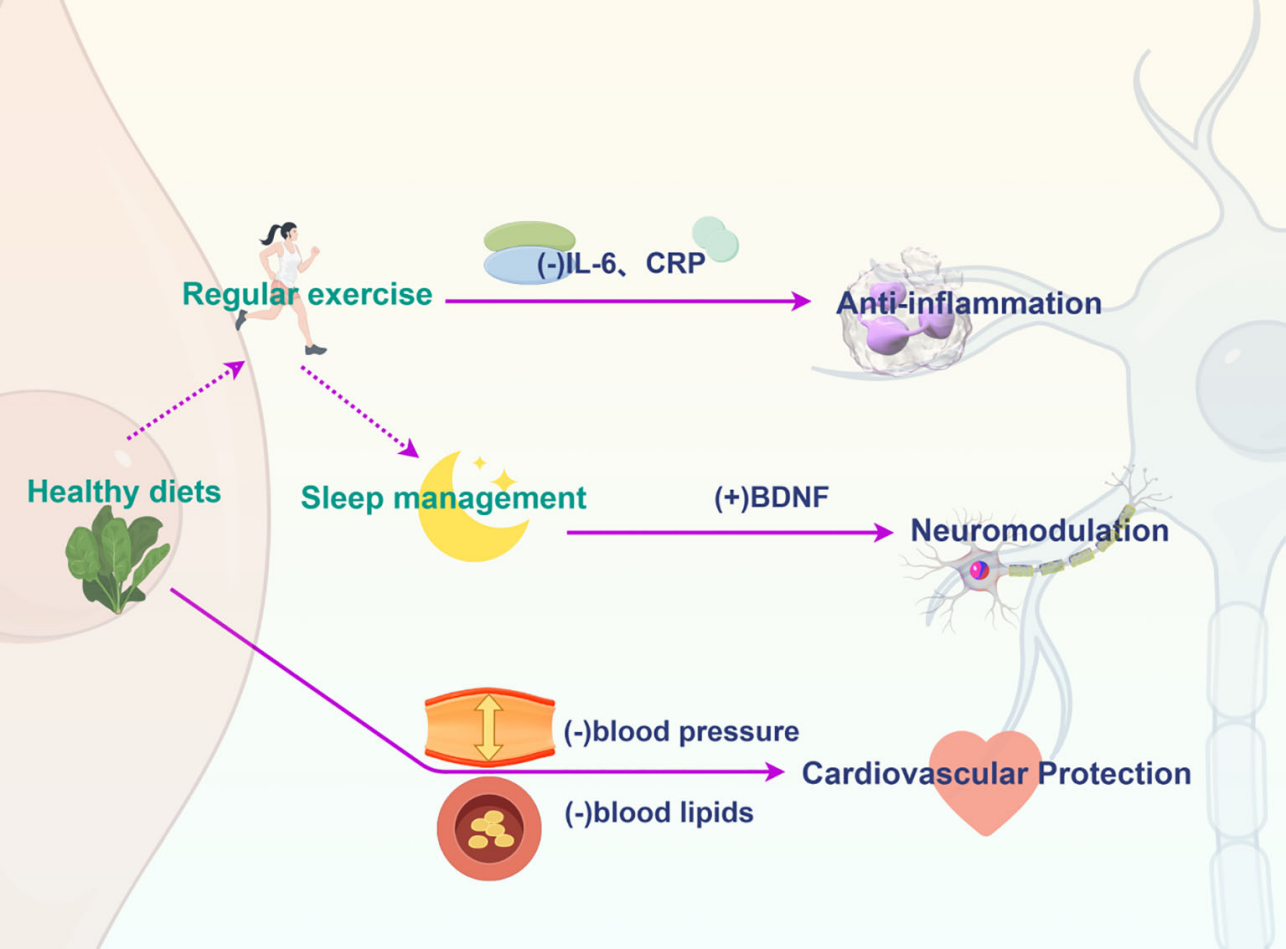
Integrated interventions such as healthy diet, regular exercise and sleep management have a simultaneous protective and ameliorative effect on CVD and depression. Solid arrows represent promoting effects, while dashed arrows represent inhibitory effects. IL-6, Interleukin-6; CPR, C-Reactive Protein; BDNF, brain-derived neurotrophic factor.

Depressive patients are also less likely to engage in physical exercise, which may be related to psychological and physical symptoms such as fatigue, loss of interest and motivation, and general bodily exhaustion. Studies have shown that depression is often associated with lower levels of physical activity, with depressed adults spending much less time on light to moderate physical activity compared to non-depressed adults (121). Depressive symptoms during hospitalization were significantly associated with reduced physical activity and regular exercise 12 months later (122). Furthermore, as depression severity increases, the time spent on both light and moderate-intensity physical activity significantly decreases. Depressed patients are more possible to lead sedentary lifestyles and fail to adhere to regular exercise routines, which is likely related to the fatigue and loss of energy commonly experienced in depression (123). The presence of depression at baseline is significantly correlated with a shift from active to sedentary behavior, and depression remains a strong predictor of sedentary behavior at both baseline and follow-up (124). The reduction in physical activity induced by depression may further lead to adverse cardiovascular outcomes. Compared to individuals who exercise, sedentary patients tend to have higher body fat percentages and more atherogenic lipid profiles, making them more prone to coronary artery atherosclerosis (125). Epidemiologic evidence suggests that low physical activity is associated with a higher prevalence of hypertension, obesity, and dyslipidemia as risk factors for cardiovascular disease (126). Furthermore, physical activity levels are strongly negatively correlated with all-cause mortality and CVD mortality (127). These findings underscore the role of reduced physical activity in promoting the development of CVD. App-based exercise interventions can be used as a supplement to traditional treatments to promote depressed patients’ Physical Activity (128). Exercise therapy can include regular aerobic exercise such as brisk walking, running, or biking, as well as strength training and flexibility exercises. A study shows that an interschool physical activity intervention significantly reduces moderate-to-severe anxiety and depressive symptoms in depressed adolescents (129). Therefore, similar physical activity programs could be implemented in school or community settings (130). One potential way to break the vicious cycle between unhealthy lifestyles and depressive symptoms in depressed patients is to use multicomponent lifestyle interventions (MLIs), which include diet, exercise, sleep, and stress management, among other integrated interventions. The effectiveness of MLIs was clarified in a study in which maintaining cognition and better adherence to nutritional and lifestyle interventions were associated with improved health-related quality of life and reduced depressive symptoms (131).The effect of the above lifestyle interventions on the improvement of CVD and depression is shown in **Figure 3**.

### 3.4 Drug side effects

During the treatment of depression, the use of antidepressants is almost inevitable. However, certain antidepressants may have adverse effects on the cardiovascular system, increasing cardiovascular risk in patients with depression. Tricyclic antidepressants (TCAs) are well-known for their cardiovascular side effects, the most common of which is orthostatic hypotension (132). This occurs due to the α-adrenergic blockade of TCAs, leading to reduced myocardial contractility and systemic vascular resistance (133, 134). For instance, amitriptyline treatment caused hypotension in patients within 10 minutes after anesthesia induction (135). Nonetheless, some studies have reported that TCAs may elevate blood pressure, which is attributed to their anticholinergic effects (136, 137). However, hypertension induced by TCAs is rare in clinical practice and can generally be ignored. The cardiovascular toxicity of tricyclic antidepressants (e.g., nortriptyline, amitriptyline) is well documented. These drugs inhibit Na+, Ca2+ and K+ channels in the cardiovascular system, often leading to life-threatening arrhythmias (138, 139), such as sinus tachycardia, bradyarrhythmias (e.g., atrioventricular block), and tachyarrhythmias (e.g., supraventricular and ventricular arrhythmias). TCAs can also cause QT interval prolongation, increasing the risk of ventricular arrhythmias (140). In comparison with SSRIs, the use of TCAs is associated with stroke, and may worsen cognitive symptoms in patients with mild cognitive impairment (141).

For patients with anxiety or depression, particularly those with comorbid CVD, SSRIs are often the first-line treatment. SSRIs selectively block the serotonin transporter, thereby inhibiting the reuptake of serotonin by presynaptic neurons and enhancing serotoninergic neurotransmission (142). Compared with other antidepressants, such as tricyclic antidepressants, SSRIs have a relatively lower incidence of cardiovascular side effects (143). Studies have shown that SSRIs benefit patients with ischemic heart disease by reducing endothelial activation, lowering C-reactive protein levels, and decreasing pro-inflammatory cytokines, as well as improving ventricular function in IHD and heart failure patients without adversely affecting electrocardiogram parameters (144–146). Clinical evidence suggests that SSRIs are generally safe for IHD patients and may even exert a cardioprotective effect. This protective effect is partly related to their ability to inhibit platelet aggregation and improve serotonin-related platelet dysfunction, thereby reducing cardiovascular mortality and morbidity (147). However, when used at high doses, SSRIs may increase the risk of arrhythmias, QTc interval prolongation, and orthostatic hypotension, even if these side effects are mild (148–150). Overall, almost all patients tolerate the cardiovascular effects of SSRIs well.

### 3.5 Psychological factors

Non-core symptoms of depression include psychological symptoms such as nervous tension and anxiety (151). A Study has shown that nearly 70% of patients with major depression also exhibit anxiety (152). Anxiety symptoms often emerge 1 to 2 years before the onset of depressive episode (153). Data from a survey showed that 45.7% of patients with major depression also suffered from one or more anxiety disorders (154). Anxiety symptoms are associated with several cardiovascular disease risk factors. Anxiety disorders increase the risk of major adverse coronary events (e.g., myocardial infarction, coronary revascularization) in patients with established cardiovascular disease (155). Paterniti’s team demonstrated that sustained high anxiety was independently linked to worsening of atherosclerosis over several years (156). A study of the U.S. population showed that anxiety was associated with a 60% increased risk of coronary heart disease in the population after controlling for common CHD risk factors (157). In addition to its direct effects, anxiety is also associated with a number of unhealthy lifestyle habits, such as smoking and drinking alcohol and lack of exercise, all of which can trigger CVD (158). Highly anxious individuals often have difficulty altering these detrimental habits. Study also shows that anxiety is associated with poor adherence to cardiac rehabilitation, particularly with regard to exercise and smoking cessation after myocardial infarction (159, 160). There is evidence that anxiety predicts subjects’ lifestyle changes and poor treatment adherence (161). Therefore, anxiety symptoms in individuals with depression may serve as a risk factor for triggering CVD.

### 3.6 Clinical research evidence

In clinical practice, depression is a standalone risk factor for cardiovascular events, heightening the likelihood of CVD and related mortality (**Table 1**). A large-scale observational study of newly diagnosed chronic heart failure patients revealed that 16.8% of patients had comorbid major depressive disorder. Among male patients with comorbid depression and anxiety, the one-year mortality risk increased by 36% (HR 1.36; 95% CI [1.28, 1.44]), while female patients experienced a 16% increased mortality risk (HR 1.16; 95% CI [1.11, 1.22]). This confirms that depression worsens survival outcomes in chronic heart failure patients and is closely associated with increased all-cause mortality (165). Furthermore, a study on patients with their first myocardial infarction and undergoing cardiac rehabilitation have found that for those with a history of depression, the hazard ratio for all-cause mortality within one year is 1.86 [1.36, 2.53], and the hazard ratio for myocardial infarction recurrence is 1.14 [1.06, 1.22] (166).

**Table 1:** Clinical evidence linking CVD and depression.

Study type	Key findings	Ref
Depression Incidence in CVD Patients	In a systematic review spanning from 1980 to 2004, major depression was identified in 19.8% (95% CI 19.1% - 20.6%) of acute myocardial infarction patients using structured interviews (N = 10,785, 8 studies). The prevalence of significant depressive symptoms based on a Beck Depression Inventory score > or =10 was 31.1% (CI 29.2% to 33.0%; N = 2,273, 6 studies).	(162)
CVD Risk in Depressed Patients	From July 1998 to December 2001, for the elderly aged 65 or above, the 15-item Geriatric Depression Scale (GDS) was used, with a GDS score of 8 or above defined as depressive symptoms. The mortality rate was followed up until March 31, 2009. Depressive symptoms were associated with mortality from coronary heart disease in men (HR 1.41, 95% CI: 1.08-1.84). Among the population aged 30 to 79 from 2004 to 2016, the World Health Organization’s Comprehensive International Diagnostic Interview Brief Form was used to assess depression. The cardiovascular mortality rate of patients with depression was 1.22 (1.04-1.44).	(5, 8)
Bidirectional Prognostic Impact	The probability of patients with acute myocardial infarction experiencing psychological distress such as depression increases significantly. About one-third of myocardial infarction patients will show depressive symptoms, while the annual incidence of severe depression in the general population is only 8.4%. At the same time, this kind of depression after myocardial infarction can significantly increase the risk of new adverse cardiovascular events in patients in the future (HR, 1.29 [95% CI, 1.15–1.43]) through multiple pathways such as reducing physical activity and lowering medication compliance, and its negative impact on cardiovascular prognosis is equivalent to that of traditional risk factors such as hypertension and diabetes.	(163)
Stroke and Depression	Using the Centre for Epidemiological Studies Depression Scale (CES-D) to assess depressive symptoms among participants in the Health and Retirement Study (HRS), the English Longitudinal Study of Ageing (ELSA), and the China Health and Retirement Longitudinal Study (CHARLS), depressive symptom trajectories were found to be significantly associated with an increased risk of stroke across all three cohorts. (CHARLS: odds ratio [OR] 2.56, 95% CI 1.62–4.04; ELSA: OR 2.96, 95% CI 1.28–6.84; HRS: OR 1.56, 95% CI 1.03–2.36), relative to the “no depression” group.	(164)

### 3.7 Genetic and multi-omics evidence

In recent years, several studies have provided genetic evidence for the bidirectional causal relationship between depression and CVD. A multivariate MR Study showed that genetic susceptibility to depression could significantly increase the risk of arrhythmia, stroke and hypertension (OR = 1.21, 1.22, 1.20, respectively), and this association was independent of confounding factors such as lifestyle. More importantly, this study found that the use of antidepressants was a key factor mediating the association between depression and CVD - the use of antidepressants was causally associated with an increased risk of all seven types of CVD, among which the association strength was the highest with atrial fibrillation and stroke (OR = 1.44) (167). Furthermore, through the analysis of the UK-Biobank database, it was found that among the three susceptibility gene scores of depression, schizophrenia and bipolar disorder, only the susceptibility gene score of depression was significantly associated with the occurrence of atrial fibrillation, coronary heart disease and heart failure, and the research results were also verified in the independent database BioVU (168). The above results further verify that genetic factors may be another important genetic mechanism linking depression and CVD, and may have potential clinical biomarker value.

## 4 Effects of cardiovascular system on depression

### 4.1 Abnormal cerebral blood flow

Adequate cerebral blood flow (CBF) is crucial for the normal functioning of the nervous system. The brain has an intrinsic ability to regulate CBF within a range of haemodynamic parameters such as arterial and intracranial pressures, thereby maintaining CBF stability (169). Under normal physiological conditions, fluctuations in cardiac output and blood oxygen levels do not significantly affect cerebral perfusion. However, abnormal reductions in CBF can exacerbate or even trigger depressive symptoms.

First, reduced CBF is a prominent feature in patients with depression. Abnormal resting CBF has been widely observed in various regions of the brain, including lower CBF in the inferior frontal gyrus, bilateral superior temporal gyri, and the anterior cingulate cortex (170, 171), alongside higher CBF in the temporal lobes, parietal lobes, bilateral thalamus, hippocampus, striatum, and posterior cingulate cortex (172). In patients with MDD, there is a significant decrease in gray matter CBF, and mood symptoms are closely correlated with reduced CBF in the dorsomedial prefrontal cortex and white matter of the frontal lobe (171). Additionally, numerous studies indicate a strong association between the onset of depression and impaired blood flow in the prefrontal-subcortical circuits. Depressed older adults with white matter high signal in medial-lateral prefrontal cortex, subcortical, and temporal lobe structures show reduced cerebral blood flow in both white and gray matter regions (173). Moreover, in recovered patients, a decrease in depressive symptoms correlates with notable rises in CBF within the left and medial prefrontal cortex, particularly the anterior cingulate cortex (174).

Secondly, individuals with depression have shown a reduced ability to regulate CBF. Two fundamental processes involved in the self-regulation of this blood flow—cerebral autoregulation (CA) and cerebral vascular response (CVR)—are markedly compromised in those experiencing depressive disorders (175). Impaired autoregulation of CBF can lead to consequences such as increased blood–brain barrier (BBB) disruption, neuroinflammation, neurodegenerative changes, and an increased risk of cerebral hemorrhage. These dysfunctions may lead to both neurological symptoms and cerebrovascular changes in depressive patients (176). Moreover, the damage to CBF autoregulation can exacerbate reduced perfusion in the prefrontal cortex, further intensifying depressive symptoms (177).

The mechanisms through which insufficient cerebral perfusion induces depression remain unclear. Both the anterior cingulate cortex and prefrontal cortex are critically involved in emotional regulation, and reduced blood flow in these regions increases the risk of mood disorders and depression (178). Additionally, in MDD patients, there is a significant reduction in absolute CBF in core regions of the default mode network (DMN), accompanied by abnormal BOLD activity, suggesting atypical neural activity at rest (179). Insufficient perfusion in the posterior cingulate cortex (a key node within the DMN) may lead to alterations in neural circuits, thus affecting emotional regulation (180). In elderly patients with depression, reduced perfusion can impair the function of specific brain regions, particularly subcortical white matter, which is prone to infarction due to limited collateral supply and impaired autoregulation (181). This damage adds to emotional and cognitive symptoms, possibly mediated by low perfusion-induced activation of microglia and subsequent neuroinflammation, resulting in a pro-inflammatory state in the central nervous system and ultimately myelin degradation and neurodegenerative alterations (182, 183). Furthermore, certain clinical features of geriatric depression, such as sleep disturbances, dizziness, and cognitive decline, may be attributed to reduced CBF. Insomnia is associated with decreased CBF in the medial prefrontal cortex, occipital cortex, parietal cortex, and basal ganglia (184). Additionally, dizziness may reflect a failure of autoregulation, indicating a loss of control over cerebral perfusion stability; conversely, impaired autoregulation and vascular reactivity may also predispose to syncope (185, 186).

### 4.2 Blood-brain barrier damage

#### 4.2.1 Introduction to the blood-brain barrier

The BBB acts as the front - line defense for the brain, shielding it from neurotoxic substances and preventing drugs from sneaking in. It consists of a single layer of endothelial cells, along with pericytes, perivascular microglia, and astrocytes (187). The endothelial monolayer is sealed by tight junctions (TJs) and adherens junctions (AJs) (188). AJs are intercellular adhesions generated by the plasma membranes of neighboring cells and are formed by the attachment of calreticulin to the actin cytoskeleton through the intermediary protein catenin (189). TJs are crucial components of the BBB. They’re made up of a variety of proteins, such as occludin, claudins, and junctional adhesion molecules. Among these, claudin - 5 stands out as the most prevalent tight junction protein and plays a pivotal role in regulating the permeability of the BBB (190). Additionally, the BBB expresses a variety of ion transporters and channels that tightly regulate the concentrations of ions such as K+, Ca2+, and Mg2+ in the brain, as well as the pH of the central nervous system (191, 192), thereby maintaining an extracellular environment conducive to the survival of neurons and glial cells.

#### 4.2.2 Effects of CVD on BBB

Recent studies exploring the interplay between CVD and BBB dysfunction have demonstrated that stroke can significantly alter BBB permeability. However, the effects of other forms of CVD on BBB integrity remain poorly understood, highlighting a critical gap in current research. BBB damage is an early pathological event in ischemic stroke, occurring before neuronal injury (193). It is characterized by the degradation of TJs and enhanced endothelial vesicular transport (194).

Following cerebral ischemia, peri-infarct neovascularization exhibits abnormally high BBB permeability due to the absence of major tight junction proteins in endothelial cells, resulting in loss of barrier integrity (195). It has been shown that local cerebral ischemia after stroke attenuates anti-inflammatory signaling and subsequently triggers the activation of resident microglia, astrocytes, and pericytes via DAMPs (196). Resident microglia are the first immune cells to be activated in the brain, probably due to the upregulation of VEGF under hypoxic conditions (197), which promotes angiogenesis and increases the permeability of the BBB (198). This alteration allows the leakage of serum proteins into the brain parenchyma, which further induces the migration of activated microglia toward the damaged area (199). These activated brain immune cells subsequently upregulate the expression of proinflammatory cytokines and chemokines that activate MMPs (200). In addition, these upregulated pro-inflammatory cytokines and chemokines recruit peripheral immune cells (e.g., neutrophils and macrophages) to the lesion, causing secondary BBB damage (201). Neutrophil infiltration is also associated with increased BBB permeability in stroke patients (202). There is evidence that inhibition of neutrophil integrin proteins prevents disruption of the BBB by reducing neutrophil recruitment to the brain after ischemic injury (203). Furthermore, neutrophils are a major source of MMP-9 in the infarcted brain region following an ischemic stroke and adds to the degradation of the BBB by hydrolyzing microvascular basement membrane proteins (204–206).

#### 4.2.3 Adverse effects of BBB disorder on depression

In depression, alterations in the BBB structure and function have been observed. Structural changes include endothelial cell damage, loosening of tight junction proteins, loss of pericyte coverage, and detachment of astrocytic end-feet (207). Functionally, peripheral immune molecules can infiltrate the brain parenchyma. Elevated levels of inflammatory markers, particularly IL-6, have been detected in the cerebrospinal fluid (CSF) of patients with depressive symptoms, suggesting BBB disruption (208). Additionally, increased BBB permeability in patients with bipolar disorder is associated with more severe depressive episodes (209).

**Figure 4:**
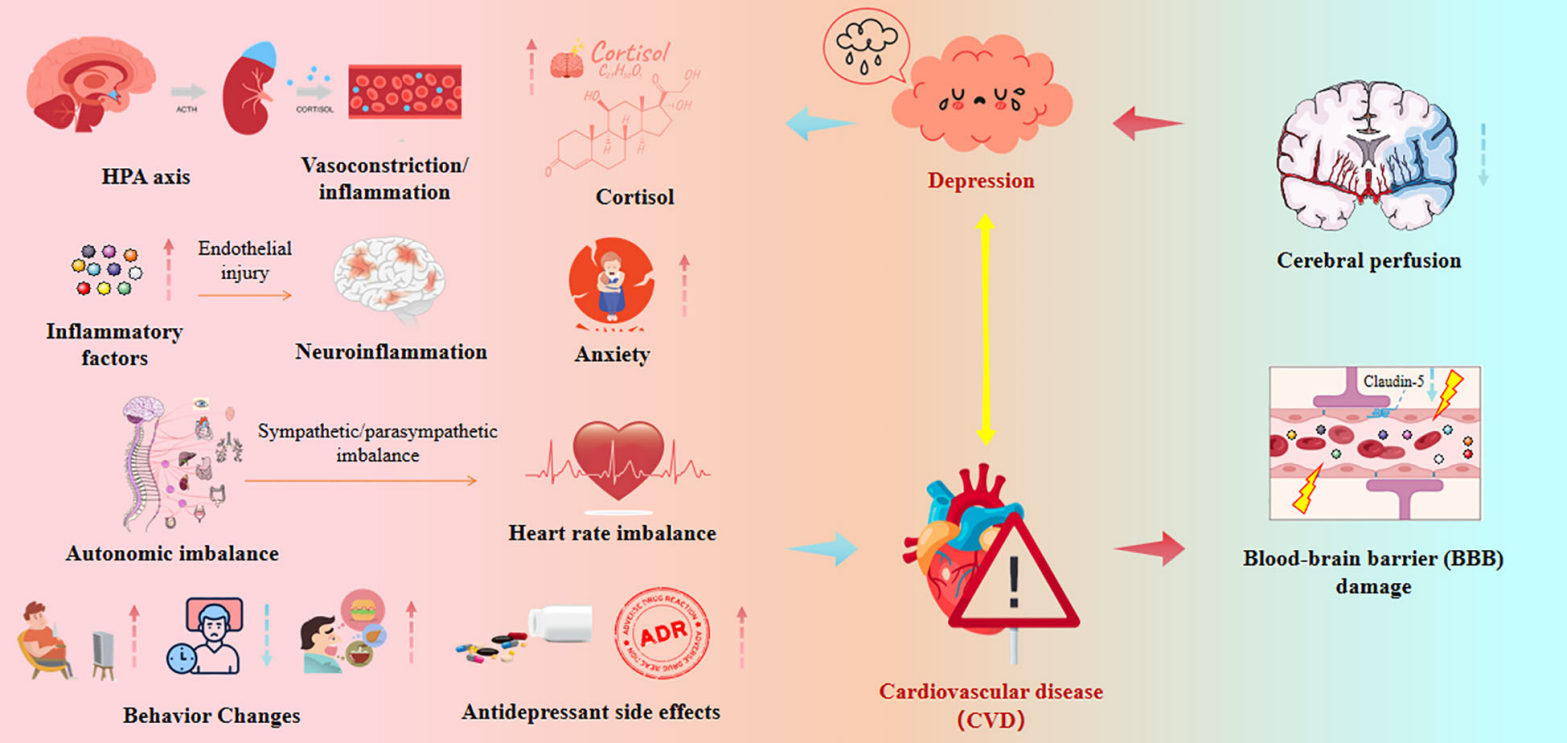
(Graphical abstract) Core mechanisms underlying the bidirectional relationship between CVD and depression. All the solid red and blue arrows represent promoting effects, while the yellow arrow represents bidirectional effects. HPA axis, hypothalamic-pituitary-adrenal axis.

In individuals diagnosed with MDD, reduced expression of the tight junction protein Claudin-5 in the hippocampus correlates with the age of onset and duration of depressive episodes (210). Furthermore, in mouse models, downregulation of Claudin-5 has been shown to induce depressive-like behaviors (211). Chronic social stress has been shown to decrease Claudin-5 expression in mice, thereby increasing BBB permeability and facilitating the entry of inflammatory mediators such as IL-6 into the central nervous system, ultimately leading to depressive-like behavior. Traumatic events have also been demonstrated to disrupt the BBB in mice, leading to the leakage of neurotrophic factors and exacerbating depressive-like behaviors (212). Moreover, The VEGF/VEGFR2 signaling pathway is crucial in the underlying mechanisms of depression, as it enhances the permeability of the BBB. This reinforces the idea that dysfunction of the BBB is a facilitating factor to the onset of depressive disorders (213). In animal stress models, BBB dysfunction, particularly involving tight junction proteins like Claudin-5, likely leads to immune cell infiltration (214). In isolated microvascular models of the sheep cerebral cortex, IL-6 has been shown to reduce the expression of Claudin-5 and Occludin proteins (214).These mechanisms are included in **Table 2** and **Figure 4**, which also incorporates the physiological mechanisms by which depression leads to cardiovascular disease.

**Table 2:** Pathophysiological mechanisms of CVD and depression.

Type of mechanism	Key molecules/pathways	Role and impact	Clinical implication	Ref
Neuroendocrine disorders	Depression→HPA axis, cortisol↑→CVD	Abnormal cortisol secretion leads to hypertension, insulin resistance, vasoconstriction, and increased CVD risk; it is also associated with depression severity.	Monitor cortisol levels to assist in assessing the risk of comorbidities and guide endocrine regulation intervention	(24, 34–36)
Inflammation response	Depression→IL-6、TNF-α、IL-1β↑→CVD	Pro-inflammatory factors promote atherosclerosis and endothelial dysfunction while inhibiting neurogenesis and exacerbating depressive symptoms.	Inflammatory factors serve as potential biomarkers for comorbidities, and anti-inflammatory treatment may synergistically improve prognosis	(73–76, 88–93)
Autonomic dysfunction	Depression→Sympathetic activity ↑, parasympathetic activity ↓→CVD	Reduced HRV, blood pressure fluctuations, increased risk of arrhythmia; associated with severity of depressive symptoms.	HRV can be used as a clinical monitoring indicator for depression to determine changes in the condition, evaluate treatment responses and predict prognosis	(41–45, 55–60)
BBB impairment	CVD→Claudin-5、VEGF→depression	Ischaemic stroke or chronic stress leads to increased BBB permeability and leakage of inflammatory factors into the center, inducing neuroinflammation and depression.	Assess the integrity of BBB. Interventions targeting Claudin-5 and VEGF may block the associated pathways of CVD and depression	(215–218)

Neuroinflammation is another key mechanism through which BBB dysfunction causes depression. This neuroinflammatory process is primarily mediated by the activation of glial cells and the elevation of pro-inflammatory cytokines (219). A disrupted BBB permits harmful neurotoxins, including cytokines and immune cells, to infiltrate the brain, which can disrupt typical brain operations. For instance, when cytokines trigger the activation of IDO, it is believed to reduce the availability of serotonin while increasing the production of the neurotoxin quinolinic acid, thereby playing a role in the onset of depression (220, 221). Uzzan and Azab (2021) showed that chronic stress-induced disruption of the BBB promotes the diffusion of peripheral inflammatory factors (such as TNF-α) into the hippocampus, which is associated with the development of MDD (222). The breakdown of BBB integrity also leads to the infiltration of peripheral CD4+ T cells and inflammatory Th17 cells into the hippocampus and prefrontal cortex. This process activates microglia, which further release pro-inflammatory cytokines and ROS, exacerbating neuroinflammation and promoting the onset and progression of MDD (223).

Additionally, the entry of pro-inflammatory factors into the central nervous system may impair the integrity of white matter structures. Damage to the BBB within the neurovascular unit could also affect cerebral perfusion, leading to structural damage of white matter tracts, as evidenced by white matter hyperintensities (224). Diffuse white matter hyperintensities are considered important factors in emotional and cognitive dysfunction and are associated with the persistence of depressive symptoms in the elderly (225). Besides neuroinflammation, BBB disruption also promotes the infiltration of circulating red blood cells into the brain parenchyma, resulting in microbleeds (226). Cerebral hemorrhage may lead to damage to dopaminergic neurons and a reduction in dopamine levels in the ventral tegmental area, which can serve as a critical mechanism for the development of depressive-like behaviors (227).

### 4.3 Clinical evidence

Depression tends to be more common in individuals with CVD than in those who are otherwise healthy (**Table 1**). A meta-analysis encompassing seven studies indicated that around 20-35% of patients following an acute myocardial infarction experienced depression (228). A recent meta-analysis covering 39 studies also revealed a significant association between cardiovascular diseases and depression. The estimated overall prevalence of depression among patients with cardiovascular diseases was 20.8%, while the prevalence of depression among patients with coronary heart disease was 19.8% and 24.7%, respectively (215). Furthermore, Among elderly individuals (mean age 82 years), participants with two, three, and four cardiovascular diseases exhibited a higher risk of depression compared to those without cardiovascular disease, with corresponding ORs (95% CI) of 1.23 (1.08–1.40), 1.50 (1.24–1.81), and 1.91 (1.42–2.56). For each additional cardiovascular comorbidity, the risk of depression increased by 15% (OR 1.15, 95% CI 1.10–1.20) (216). These findings provide clinical evidence of the elevated prevalence of depression in CVD patients.

### 4.4 Genetic and multi-omics evidence

Genetic and metabolomics studies have provided evidence for the comorbidity mechanism between CVD and depression. A meta-analysis and MR Study on cardiovascular patients have shown that different subtypes of cardiovascular diseases are significantly genetically associated with MDD. Among them, the OR of myocardial infarction and depression was 1.038, that of heart failure and MDD was 1.064, and that of hypertension and depression was as high as 1.517. These data confirmed from the genetic level the inducing effect of some subtypes of cardiovascular diseases on depression (215). In addition, a study analyzed the metabolome data of thousands of patients with major depressive disorder and found that metabolic abnormalities related to cardiovascular diseases are closely associated with depression. For instance, the metabolic characteristics of elevated very low-density lipoprotein and triglycerides and decreased high-density lipoprotein cholesterol are not only common in patients with cardiovascular diseases but also highly consistent with the “immunometabolic depression” subtype of depression. For every one standard deviation increase in triglycerides predicted by genes, the risk of depression increases by 18%, indicating that metabolic disorders caused by cardiovascular diseases may be the key bridge connecting the two (217). These findings reveal that CVD and depression share a common pathological pathway ranging from genetic predisposition to downstream metabolic disorders.

## 5 Clinical significance of bidirectional association

### 5.1 Implications for diagnosis

When diagnosing CVD and depression, it is important to focus on the bidirectional relationship between the two and conduct a thorough assessment. An increasing amount of research indicates that individuals with CVD experience depression at rates notably higher than those found in the general population. This situation not only diminishes their quality of life but can also negatively impact their prognosis. Consequently, it is advisable to implement routine depression screenings for those diagnosed with CVD. Such measures hold promise for the timely detection and effective treatment of depression, ultimately enhancing health outcomes (218). Similarly, the cardiovascular health status of depressed individuals should be given adequate attention. Depression may not only increase the incidence of CVD but also accelerate the development of multiple cardiovascular risk factors, such as hypertension, hyperlipidaemia and diabetes (229).

In the management of depression, regular assessment of cardiovascular health is advised to ensure comprehensive care. Study has shown that CVD patients should undergo depression screening by primary care physicians within a collaborative care framework. This framework typically involves a depression care manager (a dedicated healthcare professional) and a psychiatrist, who supervises and provides guidance to primary care physicians (230). The Diagnostic and Statistical Manual of Mental Disorders, Fifth Edition (DSM-5), the current widely used manual for the classification and diagnosis of mental disorders, describes in detail the diagnostic criteria for depression. According to the DSM-5, a diagnosis of depression requires that an individual exhibits at least five specific symptoms over a period of at least two consecutive weeks, including at least one core symptom (depressed mood or diminished interest or pleasure) (231). For depression screening, the Patient Health Questionnaire-2 (PHQ-2) is a quick assessment tool that takes less than a minute to complete. In CVD patients, the PHQ-2 demonstrates a sensitivity of 90% and specificity of 69% for MDD (232). In summary, to optimize overall health management, it is essential to integrate the assessment and management of both CVD and depression in clinical practice. This integrative approach can improve treatment outcomes, enhance quality of life, and ultimately reduce the incidence of cardiovascular events and depression.

### 5.2 Guidance on treatment

The treatment of depression includes three main approaches (**Table 3**): antidepressant medications, evidence-based psychotherapy such as CBT, and non-pharmacological somatic treatments including VNS and Electroconvulsive Therapy (235). Moreover, individuals with comorbid CVD and depression frequently present with metabolic disorders. Those with depression constitute a high-risk cohort for metabolic syndrome (encompassing obesity, hyperglycaemia, and other metabolic abnormalities), which itself represents a key risk factor for CVD. Consequently, managing metabolic disorders constitutes a critical intervention point for breaking the CVD-depression cycle (238, 239).

**Table 3:** Treatment strategies and clinical outcomes.

Treatment	Specific methods	Efficacy and safety	Research support
SSRIs (First-line)	e.g., Sertraline, Fluoxetine	Therapeutic effect: Reduce the levels of inflammatory cytokines and improve endothelial function; Antiplatelet effects are associated with ischemic heart disease/percutaneous coronary intervention and can reduce adverse cardiovascular events. Safety: High cardiovascular safety (low risk of prolonged QT interval); When used in combination with dual antiplatelet therapy, the risk of bleeding should be vigilant, as it may enhance the antiplatelet effect and lead to an increase in bleeding events.	(150–153)
Cognitive Behavioral Therapy (CBT)	Adjusting thought patterns and behaviors	During an average follow-up period of 94 months, patients with coronary artery disease had a 41% lower rate of first cardiovascular disease mortality and non-fatal recurrence (0.59, 95%CI 0.42-0.83). The recurrence rate of acute myocardial infarction decreased by 45%(0.55, 95%CI 0.36-0.85). Limitation: Limited efficacy for patients with severe depression or severely impaired cognitive function; The treatment outcome depends on the patient’s active participation and long-term persistence. Compliance affects the treatment effect.	(233)
Vagus Nerve Stimulation (VNS)	Electrical stimulation of the vagus nerve	Therapeutic effect: For patients with chronic moderate to severe depression, the clinical outcomes of the assisted VNS group were superior to those of the conventional treatment group (5-year cumulative remission rates were 67.6% and 40.9%, respectively). At the same time, it has a potential protective effect on cardiovascular diseases such as cardiac arrest and acute myocardial infarction (such as reducing the incidence of post-ischemic arrhythmia). Safety: There is a side effect of bradycardia. In severe cases, it may lead to cardiac arrest, and enhanced cardiac monitoring is required.	(234)
Electroconvulsive therapy (ECT)	Repair the imbalance of monoaminergic neural transmission	Therapeutic effect: suitable for treatment-resistant depression, especially for patients with severe cardiovascular diseases and intolerance to drug treatment, with rapid onset. Cardiac risk: It may temporarily affect heart rate and blood pressure, and induce arrhythmia. Cardiovascular function should be evaluated before treatment, and vital signs should be continuously monitored during treatment. Patients with severe cardiac insufficiency should use it with caution.	(235)
Lifestyle Interventions	Dietary adjustments, regular exercise, sleep management	Reduces inflammation, modulates autonomic function, and combined with medication significantly improves symptoms.	(112, 113, 236, 237)

Antidepressant medications primarily exert their antidepressant effects by increasing the concentration and availability of monoamine neurotransmitters—such as noradrenaline, dopamine, and serotonin—at neural synapses (240). In the context of treating depression in patients with or at risk for CVD, the pharmacological management often extends beyond classic antidepressants. For instance, β-blockers may be co-administered to manage comorbid conditions such as hypertension, arrhythmias, or performance-related anxiety, which are common in this population and can exacerbate cardiovascular risk. Additionally, anti-inflammatory drugs are under investigation for their potential role in mitigating the inflammatory pathways shared by both depression and CVD (241). While treating depression, patients need to be considered for the presence of comorbid cardiovascular symptoms, and measures need to be put in place to prevent possible future CVD. Currently, SSRIs are considered the first-line treatment for both cardiac and non-cardiac patients with depression (242). However, the impact of various SSRIs on cardiovascular disease risk isn’t uniform. A network meta-analysis of elderly patients with depression in China indicates that SSRIs such as escitalopram and sertraline carry a lower risk of cardiovascular adverse reactions in this population. Given that elderly depression patients frequently present with comorbid cardiovascular conditions like coronary heart disease, this study provides indirect evidence supporting the use of SSRIs in such co-morbid patients without increasing the risk of cardiovascular events (243). Current treatment guidelines recommend an acute phase of treatment lasting 8 to 12 weeks, followed by a consolidation phase of 4 to 9 months, and a maintenance phase of at least 2 to 3 years (244). For patients with CVD and depression comorbidities who also present with metabolic disorders, therapeutic options may be selected that both improve metabolic function and provide adjunctive treatment for depression or cardiovascular disease. Research indicates that the glucagon-like peptide-1 (GLP-1) receptor agonist semaglutide demonstrates significant efficacy in patients with depression and diabetes. It not only improves insulin resistance by lowering blood glucose and promoting weight loss, but also alleviates depressive symptoms through protecting synaptic plasticity and reducing hippocampal neuroinflammation (245). Another GLP-1 receptor agonist, liraglutide, can reduce endothelial dysfunction by reversing endothelial cell damage and reducing oxidative stress, thereby facilitating the alleviation of cardiovascular complications in diabetic patients (246). A latest meta-analysis shows that compared with placebo, liraglutide can reduce the risk of major adverse cardiovascular events in patients with type 2 diabetes (hazard ratio 0.86, 95% CI 0.78-0.95), including cardiovascular death, stroke and non-fatal myocardial infarction (247). In addition, the sodium-dependent glucose transporters 2 (SGLT2) inhibitor Dapagliflozin, while improving cardiac function and lowering blood sugar in patients with heart failure, can indirectly alleviate cognitive dysfunction and low mood related to depression by regulating glutamate homeostasis and the NF-κB pathway (248). For patients with dyslipidemia, statins, in addition to lowering lipids and stabilizing atherosclerotic plaques, can also play an auxiliary role in improving depression and ischemic stroke by inhibiting the activation of microglia and reducing the levels of inflammatory factors IL-1β and IL-18 in the brain (249). In clinical practice, a multi-dimensional assessment system of “metabolism - cardiovascular - mental and psychological” should be established. The metabolic indicators of patients, including HbA1c, blood lipid profile, waist circumference, cardiovascular function and depressive symptoms, should be monitored regularly, and the treatment plan should be dynamically adjusted according to the changes of the indicators. For instance, when a patient experiences aggravated insulin resistance accompanied by recurrent depressive symptoms, GLP-1 receptor agonists can be added to the existing antidepressant and cardiovascular drugs to achieve the therapeutic goals of improving metabolic disorders, stabilizing cardiovascular function and alleviating depressive symptoms (250, 251).

Cognitive behavioral therapy (CBT) is currently the most extensively researched psychological treatment approach for individuals with heart disease. CBT is considered a safe and effective method for treating depression in cardiac patients. Internet-based CBT has been shown to significantly improve depressive and anxiety symptoms in CVD patients, alongside enhancing quality of life following intervention (252). The research shows that patients with coronary artery disease who received traditional care combined with cognitive behavioral therapy had a 41% lower rate of first cardiovascular disease mortality and non-fatal recurrence during an average follow-up period of 94 months compared to those who only received traditional care (0.59 [0.42-0.83]). The recurrence rate of acute myocardial infarction decreased by 45% (0.55 [0.36-0.85]) (253).

CBT has proven to be a powerful tool in mitigating psychosocial risk factors among individuals suffering from CVD. Research focusing on patients with implantable cardioverter-defibrillators revealed that CBT led to a notable reduction in depressive and anxiety symptoms, with improvements ranging from 20% to 60% post-treatment (254). Moreover, CBT has demonstrated its efficacy in enhancing psychological well-being for those with coronary heart disease, effectively lessening feelings of depression, anxiety, and stress (255). In addition to improvements in psychological symptoms, CBT is also closely associated with the improvement of other CVD risk factors. A cardiac rehabilitation program study (256) demonstrated that CBT could increase high-density lipoprotein cholesterol levels, thereby providing protective effects against atherosclerosis. Furthermore, CBT interventions have been found to improve sleep quality and duration in CVD patients, reducing insomnia symptoms, which in turn improves cardiovascular outcomes (257).

In studies of depression associated with autonomic dysfunction, there is evidence that reduced parasympathetic responsiveness in depressed patients may adversely affect the cardiovascular system, which has provided a theoretical basis for the rise of VNS therapy in recent years (258). Research indicates that VNS may be an effective treatment for depressed patients with comorbid CVD. Compared to the control group continuing standard treatment, VNS combined with pharmacotherapy shortens the response time and reduces the risk of depressive relapse (259). Additionally, studies have pointed out that combining exercise with VNS to modulate vagal function offers a promising therapeutic strategy for the prevention and treatment of stress-induced depression and CVD (260). The 5-year study revealed that the adjunctive VNS group achieved superior clinical outcomes compared to the conventional treatment group. Specifically, the VNS group demonstrated significantly higher 5-year cumulative remission rates (67.6% vs. 40.9%) and a greater proportion of first-time remitters (43.3% vs. 25.7%) (234). In addition, after 1 month of transcutaneous vagus nerve stimulation (tVNS) treatment, the tVNS group had 24-item Hamilton Depression Scale scores were significantly lower in the tVNS group after 1 month of tVNS treatment (261). VNS also shows potential in treating a variety of CVD, including cardiac arrest, acute myocardial VNS also shows potential in treating a variety of CVD, including cardiac arrest, acute myocardial infarction, and stroke. Research indicates that chronic VNS significantly improves cardiac mechanical function and substantially reduces the incidence of ventricular arrhythmias. Concurrently, it diminishes abnormal electrical conduction blockages and restores normal myocardial repolarization in the myocardial scar margin zone. This approach thereby fundamentally reduces arrhythmias triggered by ischaemia (262). However, a major side effect of VNS is bradycardia, which can lead to cardiac arrest and can be life-threatening in severe cases (233). Therefore, intensive cardiac monitoring is recommended for patients treated with VNS (263).

In addition to common clinical treatments, lifestyle interventions are also important for managing depression comorbid with CVD. Nutrients required for nerve function, such as magnesium, folic acid, zinc, and vitamins (which are found in green leafy vegetables, legumes, whole grains, lean meats, and fish), have been shown to be beneficial for cognitive and cardiovascular health (264, 265). The Mediterranean diet is recommended for dietary management. It is rich in Omega-3 fatty acids, dietary fiber and whole grains. It can improve metabolism by promoting the production of endogenous short-chain fatty acids and enhancing insulin sensitivity. At the same time, it can alleviate depression by increasing the expression of BDNF (266). A two-year randomized trial showed that an eight-month intervention with Mediterranean dietary pattern could significantly improve depressive symptoms in patients with previous depressive episodesn (-2.42, 95%CI [-4.17, -0.67]) (267).

Furthermore, regular physical activity is widely regarded as a potent strategy for alleviating depression in individuals suffering from CVD (268). Beyond its physical benefits, exercise also combat the dual burden of depression and CVD by reducing inflammation, fine-tuning the autonomic nervous system, and improving mitochondrial efficiency in the brain and throughout the body. Study also shows that the effects of exercise are dose-dependent, with frequent moderate-to-high intensity exercise offering better outcomes for the cardiovascular system (269). The combined approach of aerobic exercise and resistance training achieves simultaneous improvements in metabolic, cardiovascular and emotional states by enhancing muscle insulin sensitivity, reducing visceral fat, and lowering serum CRP and IL-6 levels through activation of the vagus nerve-mediated anti-inflammatory pathway (270). A meta-analysis up to August 20, 2024, indicated that patients with depression who adhered to a regular training program of 150 minutes of moderate-intensity aerobic exercise and 2 resistance sessions per week experienced significant improvement in depressive symptoms (SMD=-1.39, 95%CI [-1.80, -0.96]) (271).

Smoking and excessive alcohol consumption represent shared behavioral risk factors for both depression and CVD. In patients with comorbidities, smoking not only impairs vascular endothelial function and accelerates atherosclerosis but also exacerbates depressive symptoms by disrupting neurohumoral regulation (272). Smoking cessation directly improves cardiovascular health while exerting positive effects on mental wellbeing (273). Research indicates that successful smoking cessation correlates with reduced depressive symptoms and lower rates of cardiovascular event recurrence (274). Similarly, alcohol abuse is frequently associated with mental health disorders such as depression. Moderate drinking avoids disrupting the hypothalamic-pituitary-adrenal axis, thereby preventing metabolic disorders like hypertension and hyperlipidaemia, while also reducing the likelihood of depression symptoms worsening due to alcohol-induced neurotoxicity (275, 276). Furthermore, chronic stress is a significant precipitating factor in the onset and progression of both depression and CVD. Effective stress management techniques (e.g., mindfulness meditation, cognitive behavioral therapy) have been demonstrated to reduce stress hormone levels, improve heart rate variability, and alleviate depressive and anxiety symptoms. A recent meta-analysis of patients with coronary heart disease has shown that compared with the control group, Mindfulness-based interventions significantly reduced the degree of depression (SMD = -0.73, 95%CI [-1.11, -0.35]) and perceived stress in patients, and was associated with a decrease in systolic blood pressure (SMD = -0.79, 95%CI [-1.33, -0.24]) (236). Social support networks from family, friends, or support groups can mitigate feelings of isolation and helplessness in CVD patients, decrease stress-induced inflammatory responses, thereby lowering the risk of cardiovascular events and depression, and significantly improve treatment adherence and clinical outcomes. A study involving adults with coronary heart disease or risk factors for the condition revealed that participants meeting with ten or more friends and relatives monthly demonstrated significantly higher medication adherence. Conversely, functional social support—such as the presence of a carer—had no discernible impact on adherence. This research confirms that social support networks comprising friends and relatives markedly improve treatment compliance among coronary heart disease patients (237).

## 6 Limitations

Although this review synthesizes a large amount of evidence supporting the bidirectional relationship between depression and cardiovascular diseases, some limitations should be taken into account when interpreting these findings. Much of the evidence comes from observational studies and is susceptible to confounding factors such as socioeconomic status, potential genetic predisposition or other comorbidities.

In addition, there is heterogeneity in the methods used to diagnose depression in different studies. Some people rely on structured clinical diagnoses based on DSM standards, while others use self-reported questionnaires to assess depressive symptoms. This may lead to inconsistent case classification, thereby affecting the accuracy of causal relationships.

It is worth noting that individuals engaged in healthy behaviors (such as regular exercise and an optimized diet) may be less likely to suffer from both depression and cardiovascular disease simultaneously, which creates a false association between the two situations in the observed data. Finally, the interactions among drug treatment, depression and cardiovascular outcomes have not been fully elucidated, which may confuse the observed relationships.

## 7 Conclusion

In recent years, there has been a lot of discussion on the relationship between depression and cardiovascular disease. Emerging data suggests a complex, bidirectional link between these states, mediated by a wide range of physiological and psychological mechanisms. Depression is now well acknowledged as an independent risk factor for CVD, with a major impact on the course of atherosclerosis and other cardiovascular diseases. This relationship is mediated by a variety of mechanisms, including HPA axis dysregulation and subsequent cortisol elevation, increased sympathetic nervous system activity, the release of pro-inflammatory cytokines, potential cardiovascular side effects of antidepressant medications, and the impact of psychological stressors (**Figure 4**). On the other hand, depressive patients frequently have aberrant cerebral blood flow, compromised BBB, unhealthy lifestyles, and lower medication adherence, all of which raise the risk of CVD (**Figure 4**). Therefore, effective strategies for managing the co-morbidities of depression and CVD are critical in clinical practice.

Models of collaborative care involving multiple healthcare professionals are particularly effective in populations with depression and co-morbid chronic conditions, and the creation of a multidisciplinary team that includes cardiologists, psychiatrists, primary care physicians, nurses, and psychotherapists can provide more comprehensive care for patients (277). Disease management also includes elements such as patient education, medication management, peer support, or some form of aftercare. These elements can be used as part of a disease management strategy to make patients better manage their health and reduce their risk of CVD. Cardiovascular diseases are closely related to the high prevalence of depression. Patients with heart disease often exhibit more severe depressive symptoms, which not only affect their mental health but also deteriorate clinical outcomes, such as increased mortality and delayed recovery.

Future studies could further explore the clinical applications of biomarkers in CVD and depression by developing and validating biomarkers that can predict or diagnose CVD and depression (such as CRP and leptin). In addition, gene expression changes commonly associated with CVD and depression have been investigated through genomic and transcriptomic approaches. For example, it is possible to explore the role of genes associated with CVD such as SCN5A and CXCL10 in depression and how they affect cardiovascular function and the development of depressive symptoms (278, 279). In addition, precision medicine and personalized treatment strategies are expected to be key areas of future research, aiming to achieve early diagnosis, prevention, and comprehensive treatment of depression and CVD. Changes at the genetic level can be further investigated. By analyzing a patient’s genomic information and identifying specific genetic variants associated with depression, the patient can be provided with a targeted medication regimen. This approach promises to reduce the process of trial-and-error treatment and improve treatment outcomes.

## Glossary

CVD: Cardiovascular Disease
HPA: Hypothalamic-Pituitary-Adrenal
HRV: Heart Rate Variability
BBB: Blood-Brain Barrier
PGI2: Prostaglandin I2
IFN-γ: Interferon-γ
IL: Interleukin
SSRIs: Selective Serotonin Reuptake Inhibitors
ApoB: Apolipoprotein B
ENO1: Enolase 1
AT1R: Angiotensin type 1 receptor
NADPH: Nicotinamide adenine dinucleotide phosphate
BOLD: Blood oxygen level dependent
VEGF: Vascular endothelial growth factor
DAMPs: Damage associated molecular patterns
MMPs: Matrix metalloproteinases
IDO: Indoleamine 2,3-Dioxygenase
BDNF: Brain-derived neurotrophic factor
SCN5A: Sodium Voltage-Gated Channel Alpha Subunit 5
ROS: Reactive oxygen species
CXCL10: Chemokine (C-X-C motif) ligand 10
NF-κB: Nuclear factor κ-light chain enhancer of activated B cells
TNF: Tumor necrosis factor
CRP: C-reactive protein
SMD: Standardized mean difference
